# Genome-resolved carbon processing potential of tropical peat microbiomes from an oil palm plantation

**DOI:** 10.1038/s41597-023-02267-z

**Published:** 2023-06-08

**Authors:** Aditya Bandla, Sourav Mukhopadhyay, Shailendra Mishra, Ashwin Sridhar Sudarshan, Sanjay Swarup

**Affiliations:** 1 NUS Environmental Research Institute, National University of Singapore, Singapore, Singapore; 2 Singapore Centre for Environmental Life Sciences Engineering, National University of Singapore, Singapore, Singapore; 3 Department of Biological Sciences, National University of Singapore, Singapore, Singapore

**Keywords:** Carbon cycle, Soil microbiology

## Abstract

Tropical peatlands in South-East Asia are some of the most carbon-dense ecosystems in the world. Extensive repurposing of such peatlands for forestry and agriculture has resulted in substantial microbially-driven carbon emissions. However, we lack an understanding of the microorganisms and their metabolic pathways involved in carbon turnover. Here, we address this gap by reconstructing 764 sub-species-level genomes from peat microbiomes sampled from an oil palm plantation located on a peatland in Indonesia. The 764 genomes cluster into 333 microbial species (245 bacterial and 88 archaeal), of which, 47 are near-complete (completeness ≥90%, redundancy ≤5%, number of unique tRNAs ≥18) and 170 are substantially complete (completeness ≥70%, redundancy ≤10%). The capacity to respire amino acids, fatty acids, and polysaccharides was widespread in both bacterial and archaeal genomes. In contrast, the ability to sequester carbon was detected only in a few bacterial genomes. We expect our collection of reference genomes to help fill some of the existing knowledge gaps about microbial diversity and carbon metabolism in tropical peatlands.

## Background & Summary

Peatlands occupy only 3% of the total land area but contain 600–650 Gt C^1–3^, nearly double the biomass stored in the world’s tropical rainforests^4^. Peatland ecosystems thus have a significant impact on global carbon storage, greenhouse gas emissions, and climate regulation. Although peatlands are widely distributed across the earth, some of the most carbon-dense (80–120 Gt C) peatlands exist in the tropics due to the sheer depth of peat deposits that occur within a relatively small area (0.4 million km^2^)^5^. Peatlands in South-East Asia (SEA) account for 56% of those that occur in the tropics and store nearly 77% of carbon locked in tropical peatlands globally^2^. Since 1990, tropical peatlands in SEA have been extensively repurposed (20–40% of land area) for forestry (pulpwood plantations) and agriculture (oil palm plantations)^6^, resulting in a substantial loss of sequestered carbon through peat oxidation (2.5 Gt C between 1990–2015)^5^. Peat oxidation is largely driven by microorganisms and thus our ability to predict carbon outcomes relies on identifying those with the capacity to sequester or emit carbon. In context to the data presented in this study, microorganisms refer to bacteria and archaea only.

Microorganisms in SEA tropical peat microbiomes have largely been identified and studied using marker-based approaches^7–11^. While such studies have helped uncover the high microbial diversity^9–11^ that exists within SEA tropical peatlands and its association with environmental variables^7–11^, they do not provide direct insights into microbial carbon metabolism. Identifying and understanding the role of specific microorganisms that turnover carbon in the community requires reference genomes which link microbial identity with metabolic capacity. However, genomically resolving such communities is highly challenging owing to their sheer complexity. So far, the carbon-processing potential of tropical peat microbiomes remains either genomically unresolved or poorly resolved at best, but, recent studies in temperate peatlands have shown that the functional potential of peat microbiomes can be genomically resolved using metagenomics approaches^12,13^.

**Fig. 1:**
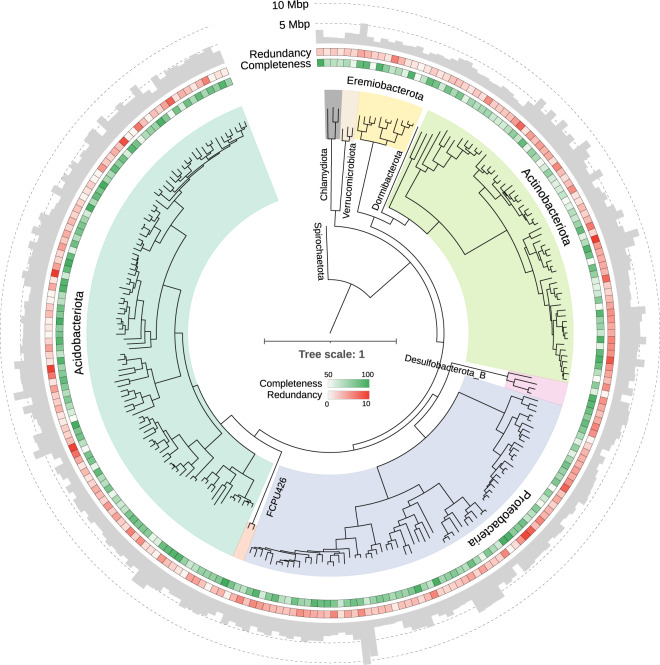
Maximum likelihood tree of bacterial species-level MAGs. The phylogenetic tree was constructed using a concatenated set of 120 conserved bacterial marker genes. Concentric rings (moving outward) represent genome completeness and redundancy. The bar plot represents the size of the MAG in Mbp.

Here, we deeply sequenced peat metagenomes proximal to and distant from oil palm trees in an oil palm plantation. Oil palm plantations on peatlands are of particular interest as they are hotspots of microbially-driven carbon emissions. Using assembly-based approaches, we reconstructed 764 sub-species level (99% gANI) metagenome-assembled genomes (MAGs) with a completeness ≥50% and redundancy ≤10% which cluster into 333 species-level (95% gANI) MAGs (245 bacterial and 88 archaeal) (Fig. 1). Of these, 38 bacterial and 13 archaeal genomes are near-complete (completeness ≥90%, redundancy ≤5%, number of unique tRNAs ≥18), while an additional 207 bacterial and 91 archaeal genomes are substantially complete (completeness ≥70%, redundancy ≤10%). The MAGs have a median size of 3.23 Mbp (range: 0.43–10.91 Mbp), median N50 of 6.34 kbp (range: 3.3–104.84 kbp) and encode a total of 2,530,130 protein-coding genes. The sub-species-level collection spans 14 different bacterial and archaeal phyla with a majority (53.1%) belonging to the phyla Acidobacteriota and Thermoplasmatota, both of which occur widely in peatlands and in acidic soils^14,15^. Within these phyla, the MAGs provide maximum phylogenetic gain for the orders UBA7540 (13 genomes; phylogenetic gain: 33%) and UBA184 (56 genomes; phylogenetic gain: 76.72%). To our knowledge, this catalogue represents the largest collection of microbial genomes from a tropical peat ecosystem.

**Fig. 2:**
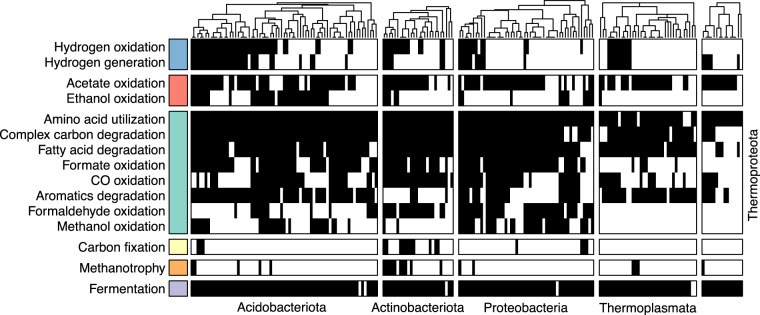
Genome-resolved carbon-processing potential of bacterial and archaeal species-level MAGs. Heatmap showing the presence (black) or absence (white) of key carbon-processing pathways across MAGs within phyla containing a minimum of ten genomes with a completeness ≥70% and redundancy ≤10%. Phylum names are shown either below or next to the heatmap slice corresponding to MAGs from the particular phylum. MAGs within each phylum are clustered based on the occurrence frequency of different pathways. Carbon-processing pathways are grouped and color-coded on the left for visual clarity.

Carbon-processing potential of MAGs was determined using a comprehensive marker-gene-based approach which integrates gene functional annotations from multiple databases such as KEGG^16^, dbCAN^17^, PFAM^18^, and TIGRFAM^19^. The ability to respire a broad spectrum of carbon substrates such as amino acids, polysaccharides, and fatty acids was widespread across both bacterial and archaeal species but the capacity to fix carbon was detected only in a few genomes (Fig. 2). Fermentative pathways which produce alcohols and organic acids such as ethanol and acetate, as well as hydrogen and carbon dioxide were also prevalent. None of the archaeal genomes encode for pathways to convert fermentative end-products into methane, however, the capacity to oxidise methane was detected in a small fraction of MAGs (34 MAGs; 10.2%) from the phyla Acidobacteriota, Actinobacteriota, Protebacteria, Desulfobacterota_B, Thermoplasmata, Thermoproteota, and Micrarchaeota. In contrast, the capacity to oxidise non-methane trace gases such as methanol (93 MAGs; 27.9%), ethanol (69 MAGs; 20.7%), hydrogen (103 MAGs; 30.9%), and carbon monoxide (177 MAGs; 53.2%) was detected in several MAGs. Interestingly, 38 of the 93 MAGs, capable of oxidising methanol belong to the phylum Acidobacteriota, members of which are not typically linked to methanol consumption.

Overall, we expect our genomes database and metagenomes to be widely useful as a reference for metatranscriptomic experiments, comparative studies, and genome-guided isolation efforts. Availability of statistics describing the prevalence of carbon-processing functions across microbial populations will help fill existing knowledge gaps about their diversity, distribution, and metabolism. This data is particularly timely as carbon emissions from repurposed tropical peatlands continue to accelerate at an unprecedented rate, posing a grave threat to our climate.

## Methods

### Sample collection

Peat samples proximal to (0.5–1 m) and distant from (≥5 m) oil palm trees were collected as part of a time-stamped fertilizer intervention experiment in an oil palm plantation located in Jambi, Indonesia (103°49ʹ 32.23ʺ E, 1°40ʹ58.24ʺ S). The plantation was considered young as age of drainage was ≤10 years^8^. The sampling location, local weather conditions and peat physiochemical parameters have been previously described^8^. The mineral fertilizer intervention involved a single application of NPK (16:16:16; P as P_2_O_5_, and K as K_2_O; 1.6–1.8 kg palm^−1^) and urea (0.5–1 kg palm^−1^) following local practices^20,21^. Peat samples were collected from four oil palm trees across two time-points before (days 1 – 2015-01-14 – and 4) and four time-points after (days 6, 7, 10, and 14) fertilizer application. All peat samples were collected from a depth 0–20 cm using an auger and flash frozen in liquid nitrogen on site.

### Metagenome sequencing and assembly

Genomic DNA was extracted from all samples using the Zymo Research Soil MidiPrep kit (Zymo Research, CA, USA). Shotgun DNA libraries were prepared from a total of 36 samples using the TruSeq DNA library preparation kit (Illumina, San Diego, CA, USA) with 2 × 250 bp chemistry, and sequenced on the Illumina HiSeq 2500 (Illumina, San Diego, CA, USA) at SCELSE (https://www.scelse.sg), Nanyang Technological University, Singapore. We generated a total of 133.7 Gbp of raw sequence data, with each sample, containing on an average, 3.8 Gbp.

Raw sequence reads were processed using Cutadapt v3.4^22^ with parameters: --error-rate 0.2, --minimum-length 75, --no-indels to remove Illumina sequence adapters. Low-quality regions from adapter-free reads were trimmed using bbmap v38.96 (https://sourceforge.net/projects/bbmap/) with parameters: trimq = 20, qtrim = rl, minlen = 75. Overall, 121.6 Gbp of sequence data were retained after quality filtering.

Samples were then assembled de-novo both individually and co-assembled in groups using MEGAHIT v1.2.7^23^ with parameters: --k-min 27, --k-max 197, --k-step 10 on 48-core compute nodes with 2T RAM. Co-assemblies of proximal and distant peat samples were performed separately by first pooling samples from each time-point and then from all the time-points. Assemblies were length-filtered to retain only contigs ≥1 kbp and renamed using the rename.sh script (bbmap v38.96) with parameters: minscaf = 1000. This resulted in a total of 10.35 million contigs (equivalent to 21.05 Gbp) with a median N50 of 1.96 kbp (range: 1.55–3.27 kbp). Read containment and across-sample contig coverage was estimated by cross-mapping each assembly against quality-filtered reads from all the samples using Bowtie2 v2.4.5^24^ with parameters: -no-unal, -X 1000, SAMtools v1.6^25^, and the jgi_summarize_bam_contig_depths script (METABAT2 v1.2.9^26^). Summaries of individual samples, assemblies, and assembled contigs ≥1 kbp are available on figshare^27^ in the files “jopf_sample_data.csv”, “jopf_assembly_summary.csv”, “jopf_single_sample_assemblies.tar.gz” and “jopf_co_assemblies.tar.gz” respectively.

### Genome binning

Genome bins were recovered from each assembly using METABAT2 v1.2.9^26^, CONCOCT v1.1.0^28^, and MaxBin2 v2.2.7^29^ with parameters: -min_contig_length 2500, all of which use a combination of differential coverage and tetranucleotide frequency information. Bins obtained using the three binning algorithms were then pooled and processed using DASTool v1.1.2^30^ with parameters: --score_threshold 0, --write_bin_evals 1, --search_engine diamond, and --write_bins 1 to achieve a unified set of non-redundant bins. This resulted in a total of 1,535 genome bins with a median size of 3.49 Mbp (range: 0.46–17.88 Mbp) and a median N50 of 6.76 kbp (range: 3.04–134.04 kbp).

### Genome refinement and dereplication

Genome bins were first refined using refineM v0.1.2^31^ with parameters: --cov_corr 0.8. Contigs were removed (a) if either their GC content or tetranucleotide frequency exceeded reference-based thresholds (98^th^ percentile) or (b) if across-sample coverage correlation was <80%. Bins were further refined using reference-based approaches implemented in MAGpurify v2.1.2^32^. Contigs were removed if they contained taxonomically-discordant marker genes, known contaminants, lacked a concordant marker gene or if they aligned poorly to conspecific genomes (when available) from the IGGdb database^32^. Refined bins with completeness ≥50% and redundancy ≤10% were designated as MAGs. Species and sub-species-level collections were generated by dereplicating the MAGs using dRep v3.4.0^33^ with parameters: -sa 0.95/0.99 --S_algorithm fastANI.

### Genome quality assessment and taxonomic classification

**Fig. 3:**
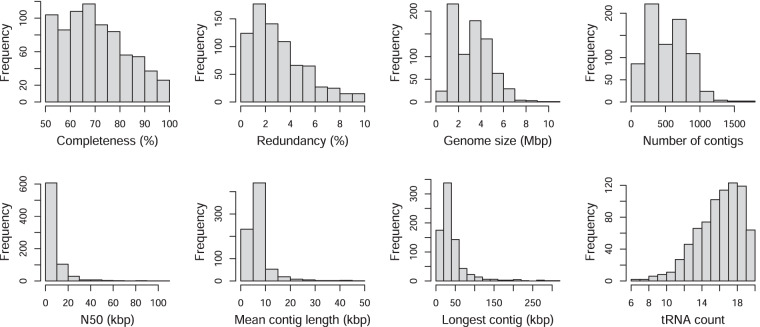
Genome statistics for the 764 sub-species-level MAGs. Histograms (from left to right, starting from the top) show genome completeness, redundancy, genome size, number of contigs, contig N50, mean contig length, length of the longest contig, and the number of tRNAs corresponding to the 20 standard amino acids identified in each MAG.

MAG statistics were estimated using CheckM v1.1.3^34^ with parameters: lineage_wf, -t 24 --pplacer_threads 1, --tab_table and are summarised in Fig. 3. Transfer RNA (tRNA) gene sequences were predicted using tRNAScan-SE v2.0.9^35^ using kingdom-specific HMM models. Taxonomic annotation was performed using GTDB-Tk v2.1.1^36–45^ and the Genome Taxonomy Database r207^46^. MAGs were classified as near-complete^31,47^ if they had completeness ≥90%, redundancy ≤5%, and ≥18 unique tRNAs, or as medium-quality otherwise. MAG statistics and taxonomic labels for the species and sub-species-level collections are available on figshare^27^ in the file “jopf_mag_quality_summary.csv”.

### Estimating phylogenetic diversity and gain

Phylogenetic diversity and gain were estimated by constructing kingdom-specific maximum likelihood trees integrating species-level MAGs from this study and reference genomes from GTDB r207^46^. Phylogenetic trees were constructed using GTDB-Tk v2.1.1^36–45^ with parameters: de_novo_wf, --bacteria/--archaea, and --outgroup_taxon p__Patescibacteria/p__Altiarchaeota. Relative taxon phylogenetic diversity and phylogenetic gain were computed using GenomeTreeTk v0.1.6 (https://github.com/dparks1134/GenomeTreeTk) with parameters: pd_clade.

De-novo trees comprising only MAGs from this study were constructed using GTDB-Tk v2.1.1^36–45^ with parameters: de_novo_wf, --bacteria/--archaea, --skip_gtdb_refs, and --outgroup_taxon p__Acidobacteriota/p__Thermoplasmatota. Trees were visualised and annotated using iTOL v6^48^. Unrooted Archaeal/Bacterial trees with and without GTDB reference genomes are available on figshare^27^ in the files “jopf_bacteria_with_gtdb_r207_refs_unrooted.tree”, “jopf_archaea_with_gtdb_r207_refs_unrooted.tree”, “jopf_bacteria_unrooted.tree”, and “jopf_archaea_unrooted.tree”.

### Functional annotation

Carbon-processing potential of the MAGs was estimated using METABOLIC v4.0^49^ which integrates functional annotations from KEGG^16^, dbCAN2^17^, PFAM^18^, TIGRFAM^19^, and custom HMMs for specific metabolic functions. Metabolic pathways were considered present if the MAG contained at least one associated marker gene or absent otherwise. Presence/absence of carbon-processing pathways in MAGs is available on figshare^27^ in the file “jopf_carbon_processing_pathways.csv”.

## Data Records

Raw metagenomes and metagenome-assembled genomes are available on NCBI BioProject PRJNA883528^50^. Datasets and data products generated from the raw data are available on figshare^27^.

## Technical Validation

MAGs reported in this study only consist of those that met the medium quality threshold or above as defined in Bowers *et al*.^51^.

## Usage Notes

Users/researchers should independently assess the accuracy of genes, contigs, and functional assignments for genomes of interest prior to downstream analysis.

## Data Availability

Open-source software packages were used to process data and generate data products. Software versions and non-default parameters are specified where required.

## References

[1] Dargie GC (2017). Age, extent and carbon storage of the central Congo Basin peatland complex. Nature.

[2] Page SE, Rieley JO, Banks CJ (2011). Global and regional importance of the tropical peatland carbon pool. Global Change Biology.

[3] Yu, Z., Loisel, J., Brosseau, D. P., Beilman, D. W. & Hunt, S. J. Global peatland dynamics since the Last Glacial Maximum. *Geophysical Research Letters* **37** (2010).

[4] Page SE, Baird AJ (2016). Peatlands and Global Change: Response and Resilience. Annual Review of Environment and Resources.

[5] Miettinen J, Hooijer A, Vernimmen R, Liew SC, Page SE (2017). From carbon sink to carbon source: extensive peat oxidation in insular Southeast Asia since 1990. Environmental Research Letters.

[6] Miettinen J (2012). Extent of industrial plantations on Southeast Asian peatlands in 2010 with analysis of historical expansion and future projections. GCB Bioenergy.

[7] Jackson CR, Liew KC, Yule CM (2009). Structural and functional changes with depth in microbial communities in a tropical malaysian peat swamp forest. Microbial Ecology.

[8] Mishra S (2014). Microbial and metabolic profiling reveal strong influence of water table and land-use patterns on classification of degraded tropical peatlands. Biogeosciences.

[9] Too CC, Keller A, Sickel W, Lee SM, Yule CM (2018). Microbial Community Structure in a Malaysian Tropical Peat Swamp Forest: The Influence of Tree Species and Depth. Frontiers in Microbiology.

[10] Dom SP (2021). Linking prokaryotic community composition to carbon biogeochemical cycling across a tropical peat dome in Sarawak, Malaysia. Scientific Reports 2021 11:1.

[11] Tripathi BM (2016). Distinctive tropical forest variants have unique soil microbial communities, but not always low microbial diversity. Frontiers in Microbiology.

[12] Woodcroft BJ (2018). Genome-centric view of carbon processing in thawing permafrost. Nature 2018 560:7716.

[13] St. James AR, Yavitt JB, Zinder SH, Richardson RE (2020). Linking microbial Sphagnum degradation and acetate mineralization in acidic peat bogs: from global insights to a genome-centric case study. The ISME Journal 2020 15:1.

[14] Belova SE (2018). Hydrolytic capabilities as a key to environmental success: Chitinolytic and cellulolytic acidobacteriafrom acidic sub-arctic soils and boreal peatlands. Frontiers in Microbiology.

[15] Sheridan PO, Meng Y, Williams TA, Gubry-Rangin C (2022). Recovery of Lutacidiplasmatales archaeal order genomes suggests convergent evolution in Thermoplasmatota. Nature Communications 2022 13:1.

[16] Kanehisa M, Goto S (2000). KEGG: Kyoto Encyclopedia of Genes and Genomes. Nucleic Acids Research.

[17] Zhang H (2018). dbCAN2: a meta server for automated carbohydrate-active enzyme annotation. Nucleic Acids Research.

[18] Mistry J (2021). Pfam: The protein families database in 2021. Nucleic Acids Research.

[19] Haft DH, Selengut JD, White O (2003). The TIGRFAMs database of protein families. Nucleic Acids Research.

[20] Woittiez LS (2019). Fertiliser application practices and nutrient deficiencies in smallholder oil palm plantations in Indonesia. Experimental Agriculture.

[21] Comeau L-P (2016). How do the heterotrophic and the total soil respiration of an oil palm plantation on peat respond to nitrogen fertilizer application?. Geoderma.

[22] Martin M (2011). Cutadapt removes adapter sequences from high-throughput sequencing reads. EMBnet.journal.

[23] Li D (2016). MEGAHIT v1.0: A fast and scalable metagenome assembler driven by advanced methodologies and community practices. Methods.

[24] Langmead B, Salzberg SL (2012). Fast gapped-read alignment with Bowtie 2. Nature Methods 2012 9:4.

[25] Li H (2009). The Sequence Alignment/Map format and SAMtools. Bioinformatics.

[26] Kang DD (2019). MetaBAT 2: An adaptive binning algorithm for robust and efficient genome reconstruction from metagenome assemblies. PeerJ.

[27] Bandla A, Mukhopadhyay S, Sridhar Sudarshan A, Swarup S (2023). figshare.

[28] Alneberg J (2014). Binning metagenomic contigs by coverage and composition. Nature Methods 2014 11:11.

[29] Wu YW, Simmons BA, Singer SW (2016). MaxBin 2.0: an automated binning algorithm to recover genomes from multiple metagenomic datasets. Bioinformatics.

[30] Sieber CMK (2018). Recovery of genomes from metagenomes via a dereplication, aggregation and scoring strategy. Nature Microbiology 2018 3:7.

[31] Parks DH (2017). Recovery of nearly 8,000 metagenome-assembled genomes substantially expands the tree of life. Nature Microbiology.

[32] Nayfach S, Shi ZJ, Seshadri R, Pollard KS, Kyrpides NC (2019). New insights from uncultivated genomes of the global human gut microbiome. Nature 2019 568:7753.

[33] Olm MR, Brown CT, Brooks B, Banfield JF (2017). dRep: a tool for fast and accurate genomic comparisons that enables improved genome recovery from metagenomes through de-replication. The ISME Journal 2017 11:12.

[34] Parks DH, Imelfort M, Skennerton CT, Hugenholtz P, Tyson GW (2015). CheckM: assessing the quality of microbial genomes recovered from isolates, single cells, and metagenomes. Genome Research.

[35] Chan PP, Lin BY, Mak AJ, Lowe TM (2021). tRNAscan-SE 2.0: improved detection and functional classification of transfer RNA genes. Nucleic Acids Research.

[36] Chaumeil PA, Mussig AJ, Hugenholtz P, Parks DH (2020). GTDB-Tk: a toolkit to classify genomes with the Genome Taxonomy Database. Bioinformatics.

[37] Hyatt D (2010). Prodigal: Prokaryotic gene recognition and translation initiation site identification. BMC Bioinformatics.

[38] Eddy SR (2011). Accelerated Profile HMM Searches. PLOS Computational Biology.

[39] Matsen FA, Kodner RB, Armbrust E (2010). V. pplacer: linear time maximum-likelihood and Bayesian phylogenetic placement of sequences onto a fixed reference tree. BMC bioinformatics.

[40] Jain C, Rodriguez-R LM, Phillippy AM, Konstantinidis KT, Aluru S (2018). High throughput ANI analysis of 90K prokaryotic genomes reveals clear species boundaries. Nature Communications 2018 9:1.

[41] Price MN, Dehal PS, Arkin AP (2010). FastTree 2 – Approximately Maximum-Likelihood Trees for Large Alignments. PLOS ONE.

[42] Ondov BD (2016). Mash: Fast genome and metagenome distance estimation using MinHash. Genome Biology.

[43] Sukumaran J, Holder MT (2010). DendroPy: a Python library for phylogenetic computing. Bioinformatics.

[44] Harris CR (2020). Array programming with NumPy. Nature 2020 585:7825.

[45] Costa-Luis, C. D. *et al*. tqdm: A fast, Extensible Progress Bar for Python and CLI. (2022).

[46] Parks DH (2022). GTDB: an ongoing census of bacterial and archaeal diversity through a phylogenetically consistent, rank normalized and complete genome-based taxonomy. Nucleic Acids Research.

[47] Almeida A (2021). A unified catalog of 204,938 reference genomes from the human gut microbiome. Nature Biotechnology.

[48] Letunic I, Bork P (2021). Interactive Tree Of Life (iTOL) v5: an online tool for phylogenetic tree display and annotation. Nucleic Acids Research.

[49] Zhou Z (2022). METABOLIC: high-throughput profiling of microbial genomes for functional traits, metabolism, biogeochemistry, and community-scale functional networks. Microbiome.

[50] Bandla A, Mukhopadhyay S, Mishra S, Sridhar Sudarshan A, Swarup S (2023). Indonesia. NCBI BioProject.

[51] Bowers RM (2017). Minimum information about a single amplified genome (MISAG) and a metagenome-assembled genome (MIMAG) of bacteria and archaea. Nature Biotechnology.

